# Modern Works on Morbid Anatomy

**Published:** 1830-01-01

**Authors:** 


### VIII.

I. ANATOMIE PATHOLOGIQUE DU CORPS HUMAIN ; OU DESCRIPTION AVEC FIGURES LITHOGRAPHIÉES DES DIVERSES ALTERATIONS MORBIDES DONT LE CORPS HUMAIN EST SUSCEPTIBLE. Par *J. Cruveilhier*, Professeur d'Anatomie, &c. First, Second, Third, and Fourth Livraison. Paris, 1828-9.

II. ELEMENTS OF GENERAL AND PATHOLOGICAL ANATOMY, ADAPTED TO THE PRESENT STATE OF KNOWLEDGE IN THAT SCIENCE. By *David Craigie*, M.D. 8vo. 1828.

[ART. I.]

THE utility of plates, in simple anatomy, may well be disputed. In general, it is not so much the colour and appearances of parts that we want to learn, as their relative position and connexion with each other. But the utility, the absolute necessity of plates to convey similitudes of morbid anatomy, cannot for a moment be questioned. In this latter study, opportunities are transitory, and the eye easily forgets that which it has but seldom seen. The most faithful verbal description fails to communicate any thing like an accurate conception of morbid structures, and is often unintelligible and even disfigured by the dominant idea of the observer. Neither are anatomical preparations without strong objections. They are altered and denaturalized by the very means of their conservation—and, at all events, must be confined to the hands of a very few, and cannot be multiplied through the instrumentality of the press. A practice the most extensive cannot furnish specimens of all diseased organs and textures. Faithfully executed plates—faithful as to form, colour, dimensions, are as lasting as Nature, and secure from the vaccillations of systems and theories. They always reproduce the same images in the mind—recal that which we have once seen—convey to others what they do not know—and leave impressions as profound as they are durable.

Eustachius deplored in the decline of his life that he had spent so much time in examining the sound structures of the body, that might have been more usefully employed in the investigation of diseased organs. Perhaps the same regret has been felt by many others since the time of Eustachius, though not so strongly expressed. At present the attention of the profession is sufficiently directed to the study of morbid anatomy, and the two works at the head of this article will shew that on both sides of the channel physicians are alive to the value of pathology. The works, however, are very different from each other. Dr. Craigie has, with infinite toil, care, and erudition, compiled a systematic verbal discussion of the various alterations which the different tissues and organs undergo in the progress of disease. It is, of course, unaccompanied by plates. It is, as is specified in the title, an *elementary* work, and consequently incapable of analysis. But we have no hesitation in characterizing it as a compilation of uncommon merit, and one which ought to form a book of reference for every practitioner, when investigating morbid structure. M. Cruveilhier's work is of a very different description—a series of cases, with faithful plates of the morbid parts, except that the diseases are classed according to the organs in which they take place. This is somewhat on the plan of Morgagni. The drawings appear to have been made as the cases occurred, and each case is generally followed by a commentary, often tedious, and uninteresting. The author is evidently placed in a situation very favourable for the study of morbid anatomy, and for the comparison of sound with diseased structures—a point of the greatest importance. It is expected that the work will be completed in about 40 livraisons or fasciculi, of which we have received four. This being the nature of the work—cases and plates—we are consequently enabled to give some account of it as it proceeds.

The first fasciculus commences with a case of transformation of the placenta into hydatidiform vesicles, of which two large plates and numerous minor figures are given.

*Case.* Madame ———, aged 24 years, previously in excellent health, became pregnant apparently soon after marriage. In the fourth month she experienced a considerable uterine loss of blood accompanied by acute pains in the loins, which recurred from time to time afterwards. In the 7th month she had strong expulsive pains, with considerable uterine hæmorrhage, in the midst of which she discharged a large mass, one half of whose surface was vesicular, the other not. A minute description of this mass, together with faithful representations of it, our author considers calculated to throw light on the theory of those singular productions known under the names of hydatid mole, vesicular mole, hydrometra hydatidica, uterine hydatids, &c. We are unable, of course, to convey a description of these plates in words: but we may briefly notice the conclusions to which M. C. has come from the anatomical examination. 1mo, He thinks this case clearly proves that placental hydatids are not a species of entozoæ, as Tyson, Goez, Cloquet and others have thought them. 2ndo, He considers it demonstrated that they are the result of a transformation, not of lymphatic vessels, as Bidloo and others have imagined; but of the sanguiferous vessels of the placenta, as may be clearly seen, both in the preparation and in the plates. Indeed Ruysch, Albinus, and Gregorini had already shewn this same transformation formerly. The mode of transformation which our author imagines, we need not stop to examine. 3tio, This vesicular transformation, he observes, is a certain, and perhaps a very frequent cause of abortion. In examining a number of expelled ova, he found a considerable proportion of the placental masses containing vesicles developed in their substance. The author has met with cases where there were diseases of the placenta of different kinds—even suppuration of the membranes, and suspects that they are more common than is generally suspected.

The subject of the third plate is a curious instance of hypertrophy of several of the cervical ganglia, one of them being upwards of two inches in length by an inch in diameter! Some of the nerves were enlarged, but not in proportion to the enlargement of the ganglia. As the case was met with in the dissecting room, and no information could be obtained respecting the history, it is needless to pursue M. Cruveilhier's conjectures as to the causes and effects of such an increase of structure in nervous ganglia.

### *Case and Plate* 4.—CANCER OF THE KIDNEY.

Vavoques, aged 53 years, entered the MAISON ROYALE DE SANTÉ, on the 9th of June, 1828, in the following condition:—emaciation without any yellow tint—extreme debility—œdema of the lower extremities—diarrhœa—thirst—smooth and pale tongue—no pain in any part of the body. On examining the abdomen, a large tumour was discovered in the left flank, indolent on pressure, and hitherto unobserved by the patient himself. It extended from the margins of the ribs of that side to the iliac fossa. At first M. C. thought it was an enlarged spleen; but the patient had not had any kind of fever for 20 years. On a more minute examination, he found the tumour to make part with the posterior parietes of the abdomen, and to be quite immoveable. The patient observed that five months previously he had been seized with a severe pain in the region of the left kidney, succeeded by hæmaturia that lasted a month, at the end of which the urine became natural. After this the diarrhœa without griping came on and continued. From these data our author came to the conclusion that the patient laboured under two distinct maladies, viz. an organic disease of the kidney, and chronic inflammation in the colon. The former might spare the life of the patient for many years—the latter must prove fatal unless remedied by art. Opiates and other medicines were directed, with farinaceous food, and blisters to the abdomen. But the diarrhœa continued—painful bleeding hæmorrhoids supervened—pulmonary affection came on—and the patient sank exhausted, with great infiltration of the lower extremities.

On dissection, the tumour was found to be an enlarged and diseased kidney. The morbid structure was various in kind, but chiefly what is called medullary or cerebriform cancer, mixed with fungus hæmatodes, and masses of scirrhus. The ureters were entirely blocked up, so that no urinary secretion took place in that side. There was ulceration of the mucous membrane of the colon.

*Remarks.* Malignant disease of the kidney is often of very difficult diagnosis. Most frequently there is no local symptom (as in the above case) but merely a gradual wasting of the flesh—a general malaise—the real seat of the malady being accidentally discovered, when not sought for. Our author relates an instance of a female aged 60 years, who came into the

Hôtel Dieu, more for rest than for any thing else, according to her own account. She was emaciated and sallow; but, in all other respects, the various functions appeared to be going on perfectly regular. After a time she demanded her discharge. Four or five days afterwards she became comatose, and sank. On dissection, the cause of death was found to be inflammation of the membranes of the brain. Our author was not a little surprised to find the right kidney greatly enlarged and completely disorganized, being converted into a state of carcinoma, the pelvis filled with black sabulous matters. The secretion of urine had not therefore been entirely suspended.

The above case gives the author an opportunity of broaching a great principle in pathology, which he promises fully to illustrate in the course of the work—namely, that "*the organic alterations, known under the name of productions, transformations, degenerations, &c. are the result of a deposit of matters secreted into the cellular tissue—hence the separation, at first, and ultimately the atrophy of the proper tissue or structure of organs or parts.*" Hence it is that the molecules of the original tissue, although dispersed, as it were, may continue to fulfil their functions to a certain extent, until they are entirely transformed into the morbid structure.

*Case.*—*Plate* 5. ACUTE NEPHRITIS—RAMOLLISSEMENT—ABSCESS AROUND THE PELVIS OF THE KIDNEY OPENING INTO ITS CAVITY.

Madame B. aged 63, of excessive embonpoint, was brought into the MAISON DE SANTÉ on the 12th September, 1828, with all the symptoms of asthma. There was much orthopnœa—face pallid—pulse somewhat fuller than natural. All that could be learnt was that the patient had been in this state for a fortnight—that she had been vomited, and twice bled. Sinapisms were applied to the feet, and an expectorating mixture was prescribed. On the 13th the oppression of breathing had entirely disappeared—yet the countenance indicated an extraordinary state of anguish and general malaise. She had vomited the whole night, and the least ingestion of drink produced reiterated nausea. The pulse was nearly natural. The medicine was discontinued, yet the sickness persisted—and the profound prostration which the patient evinced induced our author to suspect some dangerous internal disorder, the nature of which he could not divine. 15th, The abdomen was painful on pressure, especially in the left iliac region. But there was no tension—no distention—no fever. Still he thought there must be some disorganizing inflammation going forward, and therefore he ordered 20 leeches to the abdomen—sinapisms to the feet. In the evening the anguish, the vomiting, and the prostration had increased. It was now ascertained from the relations of the patient that the vomiting had continued ever since the commencement of the illness, and had been attributed to indigestion. Twenty more leeches were applied to the abdomen—blisters to the insides of the thighs—an opiate. 16th, The abdomen was still more painful; but void of tension. The anguish was inexpressible—respiration laborious—features altered—intellects undisturbed. She died in the course of the night.

*Dissection.* On opening the abdomen the peritoneum appeared sound. The exterior of the liver, spleen, stomach, and intestinal canal offered nothing particular. There was no ramollissement, and but very trifling marks of inflammation in the mucous membrane of the stomach. In short, our author was at a loss to discover any cause for death in the abdomen, and therefore proceeded to examine the thorax. Nothing was found there unhealthy! The kidneys only remained for examination. A large mass of fat was first removed from around the kidneys. In the left was found a purulent depôt in the neighbourhood of the pelvis, communicating with this cavity by means of two apertures. In the pelvis were some uric acid calculi, and a series of the same along the ureter, which was thickened and diseased. The tissue of the kidney was very much softened. The other kidney was completely atrophied.

Another investigation into this woman's history only brought to light the following facts. She had never passed any gravel. About four years previously she began to complain of dull pain in the loins, which was considered to be rheumatism. She had nausea and occasional vomiting, which were attributed to indigestion.

The author makes a judicious remark respecting the examination for a renal disease. Pressure is generally made on the abdomen; but this will seldom be sufficient. One hand should be pressed on the parietes of the loins, opposite the kidney, and with the other pressure should be made anteriorly, when enlargement or tenderness, if existing, will be detected. The above case shews that the asthmatic attacks were purely sympathetic—in other words, nervous. The vomiting, in nephritic complaints, shews the sympathy between the stomach and the kidneys. The author thinks it probable that, in this case, the calculi in the pelvis of the kidney were the primary cause of the inflammation. The case altogether is calculated to shew the difficulty of medical diagnosis, especially where renal diseases are in question. This brings us to the conclusion of the first fasciculus of the work.

The second livraison is much occupied with congenital malformations, especially club-foot, of which we can take no notice. We then come to diseases of the spleen, of which some cases and plates are given.

### *Case.*—SPLENITIS—CONCRETE PUS.

N———, aged 28 years, of strong constitution, and a cook by trade, entered the MAISON DE SANTÉ, in the following condition:—face flushed—tongue red and shining, but not furred—pulse moderately quick—perspiration abundant and constant—rheumatic pain in the left shoulder. An attentive examination of thorax and abdomen disclosed no appreciable cause for these symptoms. All that could be learnt from her history was that, a fortnight previously she had become affected with an intermittent fever, and had taken sulphate of quinine. Our author merely put her on rigid diet, and waited the event. She remained perfectly stationary for eight days. The Doctor was puzzled. He ordered some leeches to the epigastrium, not knowing what else to do. To interrogatories respecting any symptoms of ague at any particular period of the day, she always replied in the negative. All at once, in the evening of September the 11th, a formidable train of symptoms set in. She had a marked rigor, followed by a sense of suffocation, with inexpressible anguish, nausea, and vomiting of bile. These symptoms subsided in the night, and next morning she was found in her usual condition. Further examinations led to no satisfactory result. But suspecting a latent inflammation of some organ, he ordered 20 more leeches to the epigastrium—sinapisms to the feet—and blisters to the insides of the legs. *12th September.* Nearly at the same hour as before, the anguish and suffocation came on, with perpetual restlessness and bilious vomiting. The countenance became greatly altered. She said she was suffocating—she was dying. These were all the words she would speak, putting her hand sometimes to the sternum, sometimes to the epigastrium. The Doctor now concluded that the disease was a "pernicious fever," and prescribed the quinine, in doses of twelve grains a day, with eight grains in lavement. In the evening, he was agreeably surprised to find the patient much better; but she soon after had sickness and hiccup. *13th.* To day the epigastrium is very tender on pressure. The quinine to be discontinued, and leeches to be applied to the epigastrium. Quinine to be given in lavement, and to be applied to the denuded blistered surfaces. *14th.* Complained much of the pain in the left shoulder, which had disappeared for some days. The abdomen is soft and void of tenderness—tongue still very red—the pulse not very quick. The quinine en lavement to be continued. She continued in much the same state till the 19th.—the nausea and vomiting still persisting. The Doctor now suspected that some viscus was undergoing a disorganising process, and his suspicion fell on the liver, which he had often known to become studded with abscesses, and yet without any symptom during life that indicated such a state of things. The quinine was suspended—blood was drawn from the arm—amelioration of the symptoms—less sickness of stomach. In the course of the succeeding days she had warm baths—and the vomitings entirely ceased. The respiration was short, but there was no oppression complained of. She felt much better, and asked for food. Still the quickness of the pulse and the *tout ensemble* of the case indicated that there was mischief going on in some internal organ. *26th.* Jaundice supervened, and confirmed our author in his opinion that the liver was suffering, though there was no local symptom existing. During the next three days all things got worse, and the patient died on the 29th very suddenly.

*Dissection.* On accurate examination of the liver our author was surprised, and rather mortified to find it perfectly sound! The spleen was larger than natural being eight inches in diameter. Its consistence was firm, and it was covered with pseudo-membrane. The corresponding portion of diaphragm was lined with false membrane presenting numerous red patches, which were found to be blood. The false membrane being stripped off the spleen, our author was struck with the variety of colour which the surface of the organ presented. The upper extremity was marbled white and red, and the other portions were of all colours from jet black to milk white. These differences were owing to different states of disorganization. On slitting the organ into two portions, the parenchymatous structure was found to be infiltrated with concrete pus, of different hues. In only one place was there a small depôt of liquid pus. All the other organs of the body were sound—even the lungs, notwithstanding the dreadful sense of suffocation which the patient experienced.

*Remarks.* The above case affords our author much food for reflection. In the first place, here was a subacute inflammation of the spleen going on to suppuration, without any local symptom to indicate its existence. Then comes the question, was this the cause or the consequence of the periodical fever and other phenomena? M. C. concludes, at once, that the splenitis was the cause of all—the same as an inflammation of the lungs would cause symptomatic fever during its existence. All diseases of the spleen, in our author's experience, presented intermissions or remissions, which he accounts for, by the periodicity of its functions. It is true that he does not insist on the affection of the spleen being the cause of the phenomena at the beginning of an ague attack; but no doubt, he says, can exist of the influence which this organ exerts at a more advanced period of the fever. In a number of intermittents he has observed an intumescence of the spleen during each paroxysm; but the periodical afflux of blood to this organ seldom passes into inflammation. The sense of suffocation and shortness of breath, in the above case, he attributes, and with fair reason, to the adhesion of the spleen to the diaphragm, and inflammation on the surface of the latter organ.

The author makes some smart remarks on therapeutics in this place. He observes that this sanguineous fluxion is near akin to inflammatory engorgement, and demands a more active treatment than is usually employed by his countrymen. When there are symptoms of congestion or inflammation, the depletion should be prompt and decided till they disappear or until an apyrexia obtains, when the bark should be given as freely as the depletion was used boldly before.

### Reduction of the Spleen into a Pulpy Mass.

Of all organic changes that take place in this viscus, induration and softening are the most common. The former is almost always accompanied by increase of size and weight, together with fragility or cohesion of great variety in kind and in degree.

In ramollissement, the spleen never acquires such volume as in induration of the organ. The part which these splenic alterations play in the production or course of fever it is not easy to ascertain; but the author thinks that the following case contributes to the solution of the question.

*Case.* Madame N———, aged 30 years, worn down by chagrin and sorrows of every kind, became feverish on the 10th of August, but did not send for the author till the 12th, when he found her with violent headache—oppresion at the præcordia—pain in her limbs—quick pulse—red tongue—urgent thirst—great inquietude, &c. Examination of the abdomen led to no discovery.

The patient complained of pain under the false ribs on the left side. Twelve leeches to the epigastrium. 13th. Nothing remarkable. Night of the 13th very restless, with general malaise, fever, and feeling of indigestion. A new examination of the abdomen detected no tender spot; but she still pointed to the left side as the seat of her malady. Twenty more leeches to the epigastrium. The morning of the 15th was calm, but in the night the symptoms were all renewed. The day of the 16th was very good, and so was the night. 17th. All the bad symptoms returned, with faintings on the slightest movement. The complaint now assumed the appearance of a pernicious remittent fever, of which she died on the 21st of the same month.

*Dissection.* There could be but two opinions on this case, says the author. "Was it a case of gastritis? Or, was it a pernicious remittent?" The dissection was made within nine hours after death. The abdomen was distended—the stomach contracted. Its internal surface was tinged, in some places, with bile, and presented some points of injection of a darkish colour. The same might be said of the lower portion of the ileum, near the valve of the colon. There was considerable injection of the mucous membrane of the transverse arch of the colon. The colon generally was loaded with fæcal matters. The spleen was double its natural size—and so soft that it would scarcely bear handling without tearing to pieces. It was converted into a brownish pulp that almost issued forth like a liquid. The vessels of the liver were gorged, but its substance was pale. The thoracic organs were sound. The brain and spinal marrow could not be examined.

Our author thinks there cannot be a doubt that the ramollissement of the spleen commenced long before the inflammatory action in the stomach and bowels, and that it was the grand cause of the fever, and of the fatal termination of the complaint. We think it not improbable; but still there is no certainty that this disorganization of the spleen did not take place during the fever. We grant, however, that in this case the probability is in favour of M. Cruveilhier's opinion. Another case of chronic ramollissement of the spleen is given, of which we shall state the more prominent particulars.

*Case.* M. C. was called into consultation on the case of Mad. A———, whom he found in the following condition:—profound coma—immobility—complete insensibility of the upper extremities, very little in the lower—pupils contracted—respiration regular—pulse regular. The prognosis was grave. The complaint had commenced ten or twelve days previously with violent head-ache, for which leeches had been applied to the anus and epigastrium. The bleeding was copious and followed by syncope, to which the coma succeeded. Sinapisms and blisters were applied in considerable numbers. She recovered rapidly, and our author saw no more of her till fifteen days afterwards, when he was again summoned, on account of constant vomiting, which no medicine could assuage. He now learnt that the coma had disappeared in about 24 hours after the first consultation, and was followed by complete apyrexia. Some errors of regimen reproduced the febrile condition, accompanied by the vomitings above-mentioned. She had now the complexion of a person after an intermitting or remitting fever of long duration—great anxiety—small and rather quick pulse. Nothing wrong could be detected about the abdomen, nor was any pain complained of. Nothing hitherto could assuage the sickness of the stomach—and during six weeks of unceasing efforts, that is, until her death, all remedies failed to quell the gastric irritability. She died in a state of extreme marasmus. What organ was here affected? Dr. C. at first conjectured there was ramollissement of the spleen; but the protraction of the disease dissipated this idea. The liver or the stomach was then considered to be the seat of the malady. On dissection, however, the stomach was found perfectly healthy—as was also the liver. The spleen (of which a beautiful drawing is given) was greatly enlarged—of a deep brown colour—so soft that it felt like a bag full of fluid. An incision was made into it, and a very gentle pressure caused a considerable quantity of a brownish fluid to issue forth, of the consistence of thin jelly.

The foregoing case is very remarkable, on account of the intense gastric irritability which prevailed from the beginning to the end of the disease, and may fairly be said to have occasioned the death of the patient.

The third livraison commences with diseases of the lungs, and contains several beautiful plates.

### *Case.*—Pulmonary Apoplexy.

Miss Boran, aged 44 years, entered La Maison de Santé on the 31st of October, 1828, with the following symptoms:—face of a violet colour—expectoration of pure blood—respiration very quick, but she did not complain of oppression—pulse scarcely perceptible. Auscultation and percussion revealed nothing satisfactory relative to the lungs. The action of the heart was tumultuous—slight œdema of the lower extremities. It was ascertained that, 15 days previously she had violent palpitations that lasted three days successively, and left a sense of oppression about the heart. A physician prescribed an emetic, immediately after the operation of which the hæmoptysis appeared. This continued till her death on the 2nd of November.

On dissection, the lungs were found sprinkled with numerous depôts of blood, very exactly circumscribed and strongly contrasting with the perfectly sound lung in their immediate vicinity. Their size varied from that of an almond to that of a hen's egg. At first sight they appeared to be composed of blood alone; but on more accurate examination, it was found the blood was infiltrated into the cellular tissue of the lungs, and the bronchial tubes sometimes traversed these depôts. The left ventricle of the heart was in a state of hypertrophy, and the auriculo-ventricular opening of that side was diminished in size.—A very fine plate of this case is given.

The term apoplexy, originally confined to sanguineous hæmorrhage in the brain, is now transferred to depôts of blood in the lungs by a great number of accurate pathological observers. There certainly is a very great analogy between the two states. But the brain and the lungs are not the only structures in the body which are subject to these spontaneous infiltrations of blood. The skin and the subjacent cellular tissue, our author remarks, present the same phenomena, as in purpura hæmorrhagica, petechiæ, scorbutic ecchymosis, &c. Even the muscles are the seats of these depôts, as we see in those of the back among epileptics who happen to die during the paroxysm. The liver, the spleen, the uterus, heart, all offer examples of apoplexy. One of the most remarkable cases on record, was read before the Anatomical Society lately by M. Robert. All the organs, the skin, the cellular membrane, the muscles, the brain, lungs, liver, spleen, pancreas, uterus, &c. were studded with sanguineous depôts. In general, however, the infiltration is confined to a single organ, and to only a portion or portions of that organ. "What is the order of vessels concerned in this pathological state of the lungs?" asks M. Cruveilhier. Our author as well as Laennec believes that the *qualities* of the blood contribute as much towards these spontaneous hæmorrhagies as the altered condition or power of the vessels. The infiltrations seen in scurvy offer, he thinks, incontestible proofs of this doctrine. It is reasonable to suppose that a vitiated condition of the blood may weaken the vessels through which it circulates, and that thus their parietes may more readily give way than under other circumstances. But still the localization of these depôts, and the accurate manner in which their boundaries are circumscribed, would lead us to think that there must be a predisposition, that is, a weakness in the vessels of those particular localities, before the hæmorrhage occurs.

In respect to therapeutics, our author does not appear to make any reference to this local debility of vessels. "In pulmonary apoplexy," says he, "large bleedings, cutaneous and intestinal derivations, but especially large bleeding are our sole means of security." Unquestionably it is highly necessary to lessen at once the whole mass of circulation, not only by bleeding but by other evacuations also. Yet this being done, we apprehend there are some other indications of great importance to pursue—namely, to quiet the hæmorrhagic orgasm by opiates, and give strength to the vessels by such astringents as do not stimulate. The author seems indeed to admit this, though under other terms. "We must," says he, "take into consideration the state of spasm, or rather the general disturbance occasioned by the suddenness of the attack. The patient may succumb immediately from the effects of this spasm, even where the hæmorrhage is very moderate, and the portion of lung disorganized but of small extent. This spasm surmounted, the part may become re-organized, as it were, and resolution take place." Now M. Cruveilhier points out no way of overcoming this spasm or this commotion in the system, unless it is by vigorous depletion, which we venture to say will not effect the purpose.

### APOPLEXY OF THE HEART.

A female in the Salpetrière, aged 60 years, presented for several years the symptoms of dilatation with hypertrophy of the heart. She died in the usual way that people die of this class of diseases. On dissection they found the heart completely enveloped in a layer of blood, which was moulded exactly to the shape of the organ, and prolonged around the roots of the great vessels. There was also some fluid blood in the cavity of the pericardium. The layer of blood being removed, there were seen on the surface of the left ventricle several black ecchymoses, or rather sanguineous depôts, seated immediately under the attached pericardium. In one of these there was a perforation, which was evidently the source of the extravasated and concrete blood. A probe passed from this aperture into the cavity of the ventricle. But it was discovered that the extravasated blood did not proceed from the interior of the heart, as the probe had passed through the softened parietes of the depôt.

### APOPLEXY OF THE SPINAL MARROW.

We must look over several cases of gangrene of the lungs, diseases of the the liver, and aneurisms of arteries, in order to give a case of apoplexy of the spinal marrow.

*Case.* The patient was Joseph Mausard, a medical student, of slender make, and delicate constitution, but who had enjoyed good health till four or five years before the date of report, when he experienced suddenly a severe pain in the back of his neck, succeeded by some stiffness in the motions of his left leg and arm. It was considered rheumatic, sudorifics were given, and he got well in about three months. On the 10th of December, 1828, he was seized, without any apparent cause, with sharp pain in the nape of the neck abreast of the 3d or 4th cervical vertebra. In the course of a few days this pain extended along the spine with abolition of motion in the upper and lower extremities. The head was inclined towards the left shoulder, and could not be moved from this position without much pain. Mean time there was no febrile action in the system—the appetite was good—the digestion easy—respiration natural. There was now paralysis of the trunk, bladder, and rectum. The abdomen became distended—the urine was obliged to be drawn off by the catheter. In this state he entered the Maison de Santé under M. Dumeril, on the 21st of December, eleven days after the commencement of the attack. The following was the state of the case on the 1st of January, 1829, which state remained unaltered till the patient's death. Complete immobility—countenance calm—pulse regular and not weak—no head-ache—respiration natural, as were most of the internal functions—great pain in the right arm, about the shoulder-joint. All other parts were devoid of sensation as well as motion. The motions and urine were passed involuntarily—the appetite was good, the sleep was tranquil—and the spirits were even gay! He continued stationary till about the middle of January, when he began to emaciate rapidly. The integuments over the sacrum sloughed—the appetite failed—sleep could not be procured—a vomiting of blood came on, of which he died on the 18th January, forty days from the commencement of the malady.

*Dissection.* The spinal canal was laid open throughout its whole extent. The brain and its membranes were perfectly sound. The dura mater of the medulla spinalis being opened, a considerable quantity of serous fluid was found between it and the spinal arachnoid. Through this transparent fluid was seen, abreast of the 4th, 5th, and 6th cervical nerves, on the left side, a dark coloured tumour, in shape like a large almond. The left posterior half of the spinal marrow, in this part, was infiltrated with blood, and, in fact, presented a complete specimen of spinal apoplexy. The sanguineous depôt prolonged itself between the anterior and posterior roots of the cervical nerves above-mentioned, raising and separating them, altering their tint but not their continuity. There was no other visible affection of the spinal marrow externally examined; but when it was slit up, there was discovered a recent extravasation of blood in the centre of the medulla throughout its whole length, so that it was converted into a canal, as it were, full of blood. Much of this fluid was evidently of recent extravasation; but the alteration in the parietes of the canal shewed that some of it had been thrown out long before. In the thorax, the heart and lungs were sound. The stomach and intestines were lined throughout their whole length with a coating of black blood, beneath which the parietes themselves were unaltered in structure.

We had hoped to complete our account of the first four livraisons of M. Cruveilhier, but find that our limits are already exceeded, and that we must stop here for the present. The plates are very meritorious, and the work promises to be one of great value. We imagine that an enterprising publisher might find it worth his while to republish these plates, lithographied as they now are, and accompanied by a short account, in English, of the cases to which the plates refer. We shall continue our account of the work from time to time.

